# Protective effects of selegiline against amyloid beta‐induced anxiety‐like behavior and memory impairment

**DOI:** 10.1002/brb3.3599

**Published:** 2024-06-14

**Authors:** Behnam Mohamadpour, Naser Mirazi, Alireza Komaki, Hamid Shokati Basir, Abdolkarim Hosseini

**Affiliations:** ^1^ Department of Biology, Faculty of Basic Science Bu‐Ali Sina University Hamedan Iran; ^2^ Neurophysiology Research Center Hamadan University of Medical Sciences Hamadan Iran; ^3^ Faculty of Life Sciences and Biotechnology Shahid Beheshti University Tehran Iran

**Keywords:** alzheimer's disease, anxiety, oxidative stress, passive avoidance memory, selegiline, spatial memory

## Abstract

**Background:**

Alzheimer's disease (AD) is a complex and common neurodegenerative disorder. The present study aimed to investigate the potential effects of selegiline (SEL) on various aspects of memory performance, anxiety, and oxidative stress in an AD rat model induced by intracerebroventricular injection of amyloid beta_1‐42_ (Aβ_1‐42_).

**Methods:**

Oral administration of SEL at a dose of 0.5 mg/kg/day was performed for 30 consecutive days. Following the 30 days, several tests, including the open‐field, elevated plus‐maze, novel object recognition, Morris water maze, and passive avoidance learning were conducted to assess locomotor activity, anxiety‐like behavior, recognition memory, spatial memory, and passive avoidance memory, respectively.

**Results:**

The results indicate that the induction of AD in rats led to recognition memory, spatial memory, and passive avoidance memory impairments, as well as increased anxiety. Additionally, the AD rats exhibited a decrease in total antioxidant capacity and an increase in total oxidant status levels, suggesting an imbalance in oxidative‐antioxidant status. However, the administration of SEL improved memory performance, reduced anxiety, and modulated oxidative‐antioxidant status in AD rats.

**Conclusions:**

These findings provide evidence that SEL may alleviate anxiety‐like behavior and cognitive deficits induced by Aβ through modulation of oxidative‐antioxidant status.

## Graphical abstract

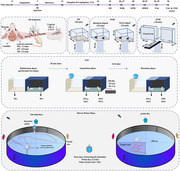

## 1 INTRODUCTION

Alzheimer's disease (AD) is a progressive neurodegenerative disorder that affects approximately 50 million people worldwide (Iraji et al., 2020). It is the most common form of dementia and the fifth leading cause of death in individuals over 65 years old (Huang et al., 2020). AD is characterized by memory and learning impairments, cognitive decline, and behavioral symptoms that interfere with daily functioning (Thies et al., 2013). In addition to progressive impairment of cognitive function, individuals with AD frequently experience various non‐cognitive disturbances, such as depression and anxiety, which worsen as the disease advances (Ghaderi et al., 2022). The disease is believed to result from a combination of genetic, environmental, and lifestyle factors (Bulgart et al., 2020). The cellular phase of AD coincides with an imbalance between the production and clearance of amyloid‐beta (Aβ) in the brain, inducing the spread of neurofibrillary tangle formation in the brain, which contains hyperphosphorylated tau protein (Meghana et al., 2023; Scheltens et al., 2021). Oxidative stress has been implicated as a key pathological factor in AD, connecting various hypotheses and mechanisms of the disease and causing neuronal damage in the cortical and hippocampal regions of the brain through different pathways (Bai et al., 2022; Ghai et al., 2020; Pérez‐Areales et al., 2020). Therefore, strategies aimed at restoring the oxidative‐antioxidant balance and reducing Aβ accumulation in the brain are crucial for preventing or treating the disease (Y. Zhang et al., 2023). Previous studies have highlighted that Aβ peptides disrupt the oxidative‐antioxidant balance, impair learning and memory, and consequently lead to cognitive decline (Ahmadi et al., 2021).

It has been shown that excessive expression of monoamine oxidase‐B (MAO‐B) in astrocytes has been implicated in the improper breakdown of monoamine neurotransmitters such as dopamine, norepinephrine, epinephrine, and serotonin. This abnormal breakdown can lead to an increased production of free radicals. These processes are believed to contribute to the neurodegenerative processes observed in AD (Rahman, 2007). These findings suggest that the inhibition of MAO‐B through the use of drugs may offer a new strategy to alleviate the pathological symptoms of AD (Behl et al., 2021). Selegiline (SEL) is used as an adjunctive therapy in the management of patients with Parkinson's disease and as a treatment for major depressive disorder in adults. It acts as a selective inhibitor of MAO‐B, blocking the metabolism of dopamine and preventing its degradation, thus increasing dopamine levels and improving motor symptoms in patients (Moore & Saadabadi, 2022). It has been hypothesized that SEL inhibits the reuptake of synaptic dopamine and prolongs its activity, potentially aiding in the improvement of dopaminergic neuron function (Nagatsu & Sawada, 2006). Furthermore, the neuroprotective effects of SEL may be attributed to increased production of neurotrophins such as nerve growth factor and brain‐derived neurotrophic factor, which protect neurons against inflammatory processes. These inductions and activations of several anti‐oxidative stress and anti‐apoptotic factors may help maintain brain tissue integrity (Nagatsu & Sawada, 2006). Given SEL's capability to increase levels of monoamine neurotransmitters, particularly dopamine (Ishikawa et al., 2019), it offers a potential avenue for improving cognitive deficits associated with AD. However, minimal research has been conducted on the effect of SEL in animal models of AD (Pazini et al., 2013; Tsunekawa et al., 2008). To the best of the authors’ knowledge, only two studies show that SEL can improve cognitive deficits in animal models of AD, possibly through modulation of cholinergic, dopaminergic, and monoaminergic systems (Pazini et al., 2013; Tsunekawa et al., 2008). Conversely, this study aimed to examine the effects of SEL on behavioral functions using a wide range of standard behavioral tests, including open‐field (OF), elevated plus‐maze (EPM), novel object recognition (NOR), Morris water maze (MWM), and passive avoidance learning (PAL), in an Aβ_1‐42_‐infused AD rat model. The main focus of this study was to evaluate oxidative stress as a potential underlying mechanism to determine different aspects of the possible protective effects of SEL on AD‐induced behavioral deficits.

## 2 MATERIALS AND METHODS

### 2.1 Animals and experimental design

In the present study, adult male Wistar rats weighing 200–220 g were used. They had free access to food and water and were kept under standard laboratory conditions with alternating light‐dark cycles of 12 h each. Animal care and experimental protocols were performed in accordance with the NIH guidelines and were approved by the Ethics Committee of Bu‐Ali Sina University‐Hamadan (ethical code IR.BASU.REC.1400.001).

The rats were acclimated to the laboratory conditions for 1 week and were then randomly divided into five groups of eight animals each as follows:

1. Control group: Rats received 5 mL/kg/day of normal saline via oral gavage (P.O.) for 30 days.
2. PBS group: Rats received a stereotaxic U‐ICV injection of 5 µL/rat of phosphate‐buffered saline (PBS) plus normal saline (5 mL/kg/day; P.O. for 30 days).
3. SEL group: Rats received selegiline (0.5 mg/kg/day; P.O. for 30 days).
4. AD group: Rats received a stereotaxic U‐ICV injection of 5 µL/rat of Aβ_1‐42_ (1 µg/μL) plus normal saline (5 mL/kg/day; P.O. for 30 days).
5. AD + SEL group: Rats received selegiline (0.5 mg/kg/day; P.O. for 30 days) after the U‐ICV injection of 5 µL/rat of Aβ_1‐42_ (1 µg/μL).

**FIGURE 1:**
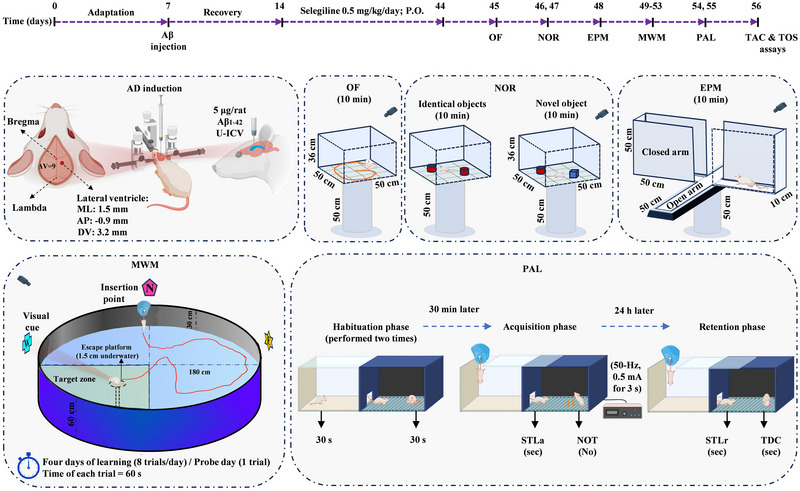
After a week of acclimation, the rats in the AD and AD + SEL groups received A 5‐µL solution of Aβ_1‐42_ (1 mg/mL). SEL at a dose of 0.5 mg/kg/day was then orally administered to the SEL and AD + SEL groups for 30 days. Subsequently, anxiety, recognition, spatial, and passive avoidance tests were performed. At the end of the experiment, plasma levels of total antioxidant capacity (TAC) and total oxidative stress (TOS) were measured. EPM, elevated plus‐maze; MWM, Morris water maze; NOR, novel object recognition; NOT, number of trials to reach learning; OF, open‐field; PAL, passive avoidance learning; STLa, step‐through latency in the acquisition trial; STLr, step‐through latency in the retention phase; TDC, time spent in the dark compartment.

The SEL solution was administered orally for 30 consecutive days. The dose of SEL was selected based on previously published information (Arib et al., 2010; Bickford et al., 1997; Carrillo et al., 1993; Carrillo et al., 1996; Muralikrishnan et al., 2003; F. Zhang et al., 2015). Figure 1 shows the timeline of the experimental procedures. The behavioral tests of the rats (*n* = 8) were evaluated by employing OF on day 45, NOR on days 46 and 47, EPM on day 48, MWM during days 49–53, and PAL on days 54 and 55. At the end of the study on day 56, the rats were euthanized for biochemical assessments (*n* = 8).

### 2.2 AD induction

A 5 µL solution of Aβ (1 mg/mL) was administered to induce AD in rats. Therefore, Aβ peptide_1‐42_ rat (product No./SKU SCP0038‐1MG, Sigma Aldrich) was dissolved in PBS solution. Before U‐ICV injection, the Aβ solution was incubated at 37°C for 4 days. Amyloid fibrils are produced during this process, which are neurotoxic (Basir et al., 2024; Lorenzo & Yankner, 1994).

For AD induction, each rat was anesthetized by intraperitoneally injection of ketamine (100 mg/kg) and xylazine (10 mg/ kg) and positioned in a stereotaxic apparatus (Stoelting Co.). The head was shaved and a midline sagittal incision was made in the scalp. A tiny hole was drilled carefully up to the level of the dura mater in the skull over the ventricular area (M/L: 1.5 mm laterally from the bregma and A/P: −0.9 mm posteriorly from the Bregma). Hamilton syringe needle was slowly directed down to beneath the surface of the cortex for the U‐ICV injections, into the right lateral ventricle at a depth of 3.2 mm based on the Paxinos brain atlas (Paxinos & Watson, 2006). A 5 µL of Aβ solution was administered at a rate of 0.5 µL/min. The PBS group received 5 µL of PBS (10 mM), the same volume as the Aβ injection (Figure 1).

### 2.3 Open‐field test

The test utilized an OF device positioned 50 cm above the room floor. This device consisted of a wooden box measuring 50 cm in length, 50 cm in width, and 36 cm in height. The box had opaque walls, which served to isolate the interior from the external environment and minimize the impact of environmental factors. The video tracking software (Borjsanat Co) analyzed the locomotor activity of the rats inside the box using a camera placed on top of the box. The rats were transferred to the testing room 30 min before the start of the experiment to acclimate to the environment (Gholipour et al., 2022). Each rat was placed in the center of the box to freely explore for 10 min. The inner wall of the box was cleaned with alcohol after each trial.

### 2.4 NOR test

The experimental rats were placed in a box with dimensions of 50 cm length × 50 cm width × 36 cm height in three phases: (1) Adaptation to the empty chamber, (2) familiarization phase with two identical objects, and (3) test phase (test day). On the first day, each animal was placed inside the empty chamber for 10 min to familiarize themselves with the new environment. On the same day, at a 6‐h interval, two identical cylinders were placed at a distance of 5 cm in two corners of the chamber, and each rat was placed inside the chamber for 10 min. On the second day, one of the cylinders was replaced with a cube object. The rat behavior was recorded using a camera. The exploration ratio, as a measure of the time spent exploring the new object, was calculated by dividing the duration of exploring the new object by the total duration of exploring both old and new objects (Ahmadi et al., 2021).

### 2.5 EPM test

The EPM device is located 50 cm above the floor and consists of two enclosed arms and two open arms facing each other. The two open arms have dimensions of 50 cm × 10 cm, are wall‐less, and white, while the two enclosed arms are 50 cm × 10 cm × 50 cm with walls and black. Each rat was initially placed in one of the open arms while facing the center of the maze, and its behavior was recorded for 10 min. During this time, the number of entries into the open arms and the duration of stay in the open arms were recorded to assess anxiety‐like behavior (Karimi et al., 2019). The ratio of entries into the open arm was calculated by dividing the number of entries into the open arm by the total number of entries into both types of arms.

### 2.6 MWM test

The MWM is a cylindrical tank with a black inner coating, 180 cm in diameter and 60 cm in height, filled with water at a temperature of 26 ± 2°C up to a depth of 30 cm. The maze has four imaginary quadrants: northeast, southeast, northwest, and southwest. A hidden circular platform with a diameter of 10 cm is located 1.5 cm below the water surface. The platform is placed in the center of one of the imaginary quadrants. This hidden platform is the only escape route for the animal from the water reservoir. The room containing the water maze has external cues such as visual symbols. A camera installed above the reservoir tracks and detects the animal's movement. To familiarize the rats with the maze, they were placed in the platform‐free reservoir for 1 min and 24 h before training to swim.

During the 4 days of training, in each training trial, the animal was randomly released from one of the four main points of the reservoir (north, south, east, and west) into the water. The rat swam until it found the hidden platform and climbed onto it. If the platform was not found within 60 s, the animal was guided to it and allowed to stay on it for 30 s. After the final training session, the rat was dried with a towel and returned to its cage. The rats underwent training for 4 consecutive days, with each rat receiving eight trials per day divided into two blocks of four trials. The learning progress of the animals was measured based on the time spent and distance traveled in the maze to find the platform (Ramezani et al., 2020).

During the memory test on day 5, the hidden platform was removed and the mice were released into the water from a single starting point, and their movements (percentage of time spent in each quadrant of the maze) were recorded for 60 s. The time spent in the target quadrant was evaluated as an indicator of spatial memory (Rashno et al., 2022).

At the end of the memory test, the visual acuity of the animals was also evaluated. In this stage, the platform was raised above the water surface and made visible by a white polystyrene. The rat was then randomly released into the water and given 60 s to find the platform. If there was a visual impairment, the rat was excluded from the statistical analysis (Zarrinkalam et al., 2016).

### 2.7 PAL test

The PAL test was performed using a shuttle box apparatus, which consisted of two compartments, light and dark, each measuring 23 cm × 23 cm × 30 cm. The two compartments were connected by a guillotine door measuring 7 cm × 9 cm. The floor of both compartments had metal rods with a diameter of 1 mm and spaced 1 cm apart. Electric shocks were delivered to the animal's feet through these rods in the dark compartment using a stimulator device. Initially, each animal was placed in the light compartment for 30 s to familiarize themselves with the apparatus. Due to the natural preference for the dark environment, the animal would enter the dark compartment. After 30 s of being in the dark compartment, the animal was removed and after 2 min, this process was repeated. If the animal avoided entering the dark compartment within 120 s, the learning test was terminated; otherwise, after complete entry into the dark compartment, the animal received a mild electric shock (50 Hz, 0.5 mA for 3 s) through the floor rods. After 30 s, the animal was removed from the dark compartment and the process was repeated after 2 min. The number of trials in which each animal received a shock before avoiding entry into the dark compartment (NOT) was considered an indicator of animal learning. For the memory test, 24 h after the learning test, the animal was placed in the light compartment and after 30 s, the guillotine door between the two compartments was opened and the behavior of the animal was evaluated for 300 s. During this time, the delay in entering the dark compartment step‐through latency in the retention phase (STLr) and the time spent in the dark compartment (TDC) were measured and compared between groups.

### 2.8 Biochemical assay

Blood samples were collected from the hepatic portal vein of each rat after anesthesia with a mixture of ketamine (100 mg/kg) and xylazine (10 mg/kg). Each sample was centrifuged at 3500 rpm for 20 min, and the clear plasma was divided into 100 µL aliquots and stored at −80°C until use. The measurement kits for total oxidant status (TOS) and total antioxidant capacity (TAC) (Kiazist Life Sciences) were used according to the manufacturer's protocols to calculate the values of oxidative and antioxidant biomarkers, respectively.

### 2.9 Statistical analysis

Data were analyzed and plotted using GraphPad Prism software, version 9 (GraphPad Software). Normality of data was assessed using the Shapiro–Wilk test (*p* > .05). Analysis of variance were performed, and in case of significance, post hoc analysis was conducted using the appropriate test (based on the result of Bartlett's test for equality of variances between groups). The MWM test was compared using two‐way and one‐way analysis of variance. One‐way analysis of variance followed by Tukey's post hoc test was performed for OF test, NOR test, and EPM test, and the results were presented as mean ± standard deviation (mean ± SD). The shuttle box test was analyzed using the Kruskal–Wallis test followed by Dunn's post hoc test, and the results were presented as medians, interquartile ranges, maximums, and minimums. A significance level of *p* < .05 was considered in all statistical analyses.

## 3 RESULTS

### 3.1 The effects of SEL on the OF test in AD rats

**FIGURE 2:**
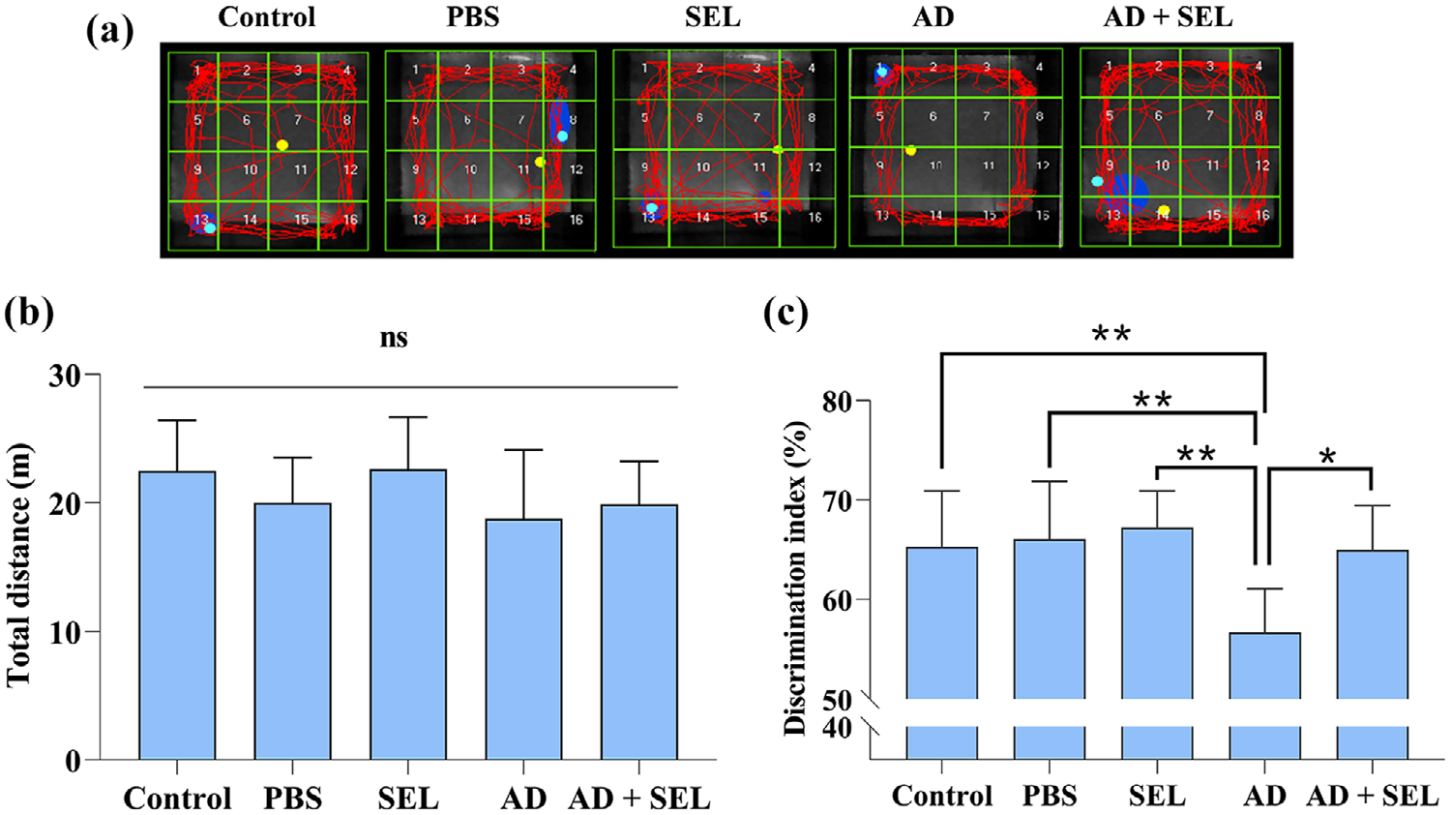
Samples of recorded activities of rats in the OF test (a). Effects of selegiline on locomotor activity in the open‐field test (b) and the discrimination index of the novel objective recognition (c) of rats. Data are presented as means ± SD of eight animals per group (one‐way analysis of variance). ns, no significance; *p < .05, **p < .01, ***p < .001. AD, Alzheimer's disease; PBS, phosphate‐buffered saline; SEL, selegiline.

Samples of recorded activities in the OF test are shown in Figure 2a. The one‐way analysis of variance did not show a significant difference in the distance traveled by the rats in the OF test (*F* (4, 35) = 1.40; *p* = .25; Figure 2b). In other words, Alzheimer's surgery and SEL treatment did not affect locomotor activity.

### 3.2 The effects of SEL on the NOR test in AD rats

The one‐way analysis of variance showed a significant difference in the novel object exploration ratio between groups (*F* (4, 35) = 5.98; *p* < .001; Figure 2c). Based on this, Tukey's test showed a significant decrease in the AD group compared to the control group (*p* < .01). Additionally, the AD + SEL treatment group showed a significant increase compared to the AD group (*p* < .05).

### 3.3 The effects of SEL on the EPM test in AD rats

**FIGURE 3:**
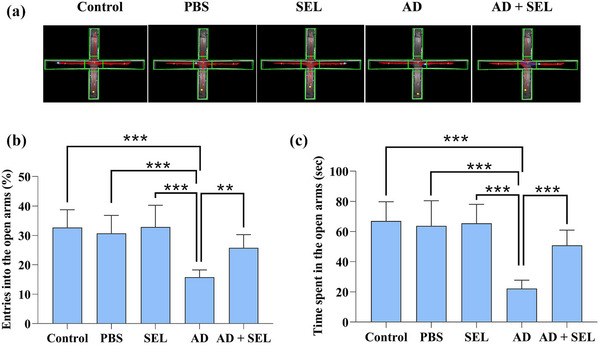
Samples of recorded activities of rats in the elevated plus‐maze (EPM) test (a). The effects of selegiline administration on the open arms entries (b), time spent in open arms (c) of the elevated plus maze Test. Data are presented as means ± SD of eight animals per group (one‐way analysis of variance, Tukey's post hoc test). **p < .01, ***p < .001. AD, Alzheimer's disease; PBS, phosphate‐buffered saline; SEL, selegiline.

Samples of recorded activities in the EPM test are shown in Figure 3a. The one‐way analysis of variance showed a significant difference in the percentage of entries into open arms between groups (*F* (4, 35) = 13.22; *p* < .001; Figure 3b). Based on this, Tukey's test showed a significant decrease in the AD group compared to the control group (*p* < .001). Additionally, the AD + SEL treatment group showed a significant increase compared to the AD group (*p* < .01).

The one‐way analysis of variance showed a significant difference in the time spent in open arms between groups (*F* (4, 35) = 19.21; *p* < .001; Figure 3c). Based on this, Tukey's test showed a significant decrease in the AD group compared to the control group (*p* < .001). Additionally, the AD + SEL treatment group showed a significant increase compared to the AD group (*p* < .001).

### 3.4 The effects of SEL on the MWM test in AD rats

**FIGURE 4:**
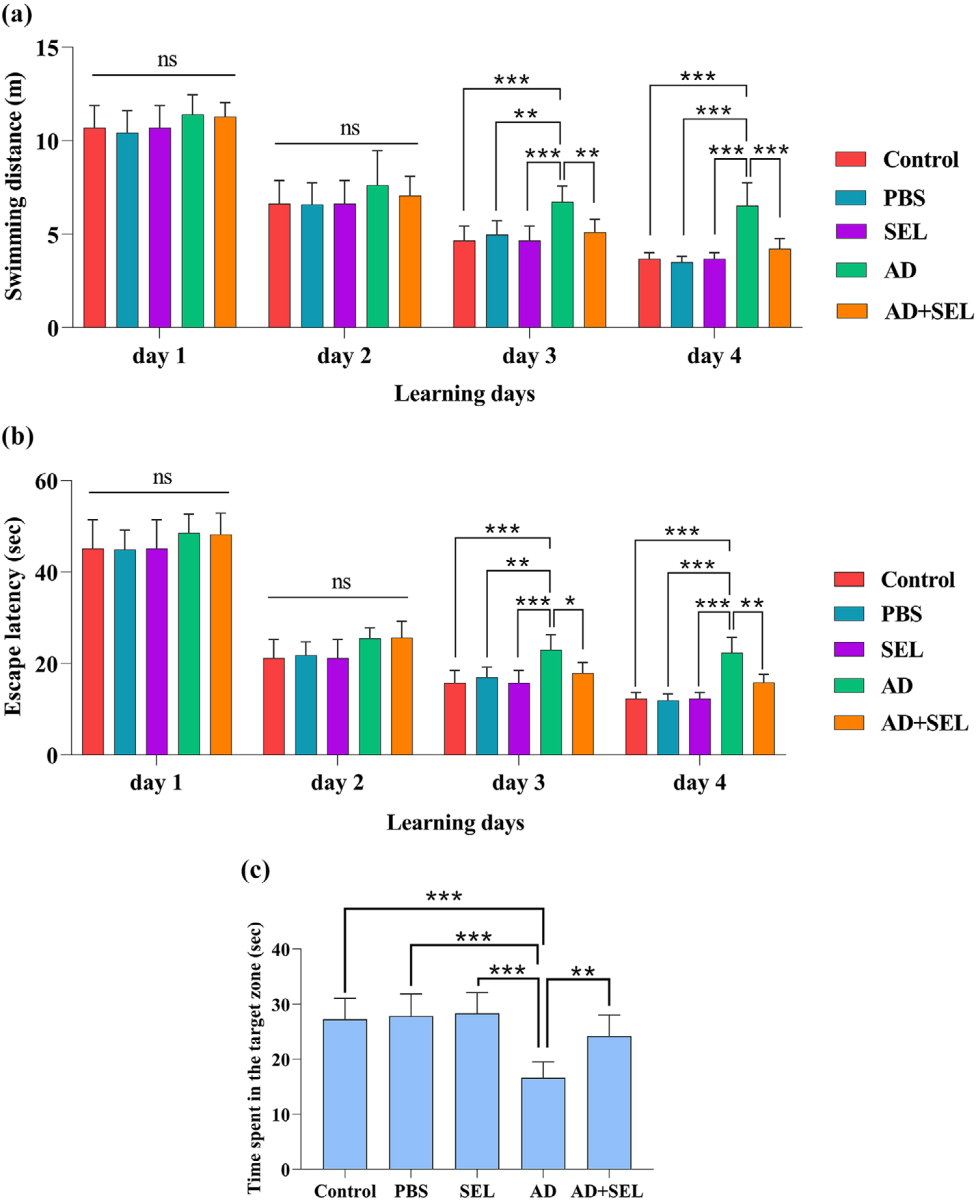
The effects of selegiline administration on the swimming distance (a) and escape latency (b) to the hidden platform in the training trials, and time spent in target zone (c) in the Morris water maze Test. Data are presented as means ± SD of eight animals per group (two‐ and one‐way analysis of variance, Tukey's post hoc test). ns, no significance; *p < .05, **p < .01, ***p < .001. AD, Alzheimer's disease; PBS, phosphate‐buffered saline; SEL, selegiline.

The two‐way analysis of variance showed a significant difference in swimming distance during the 4‐day training (day: F _[3, 28]_ = 238.6; *p* < .001; treatment: F _[4, 112]_ = 18.90; *p* < .001; Figure 4a). Following this, Tukey's test showed a significant increase in the AD group compared to the control group on day 3 (*p* < .001) and day 4 (*p* < .001). Oral administration of SEL resulted in a significant decrease in the AD + SEL treatment group compared to the AD group on day 3 (*p* < .01) and day 4 (*p* < .001).

The two‐way analysis of variance showed a significant difference in the time to reach the hidden platform underwater during the 4‐day training (day: F _[3, 28]_ = 419.4; *p* < .0001; treatment: F _[4, 112]_ = 22.09; *p* < .001; Figure 4b). Following this, Tukey's test showed a significant increase in the AD group compared to the control group on day 3 (*p* < .001) and day 4 (*p* < .001). Oral administration of SEL resulted in a significant decrease in the AD + SEL treatment group compared to the AD group on day 3 (*p* < .01) and day 4 (*p *< .01).

The one‐way analysis of variance showed a significant difference in the results of the probe day analysis (*F* (4, 35) = 14.01; *p* < .001]. Based on this, the AD group had a significant decrease compared to the control group (*p* < .001). Additionally, the AD + SEL treatment group showed a significant increase compared to the AD group (*p* < .01; Figure 4c).

### 3.5 The effects of SEL on the PAL test in AD rats

**FIGURE 5:**
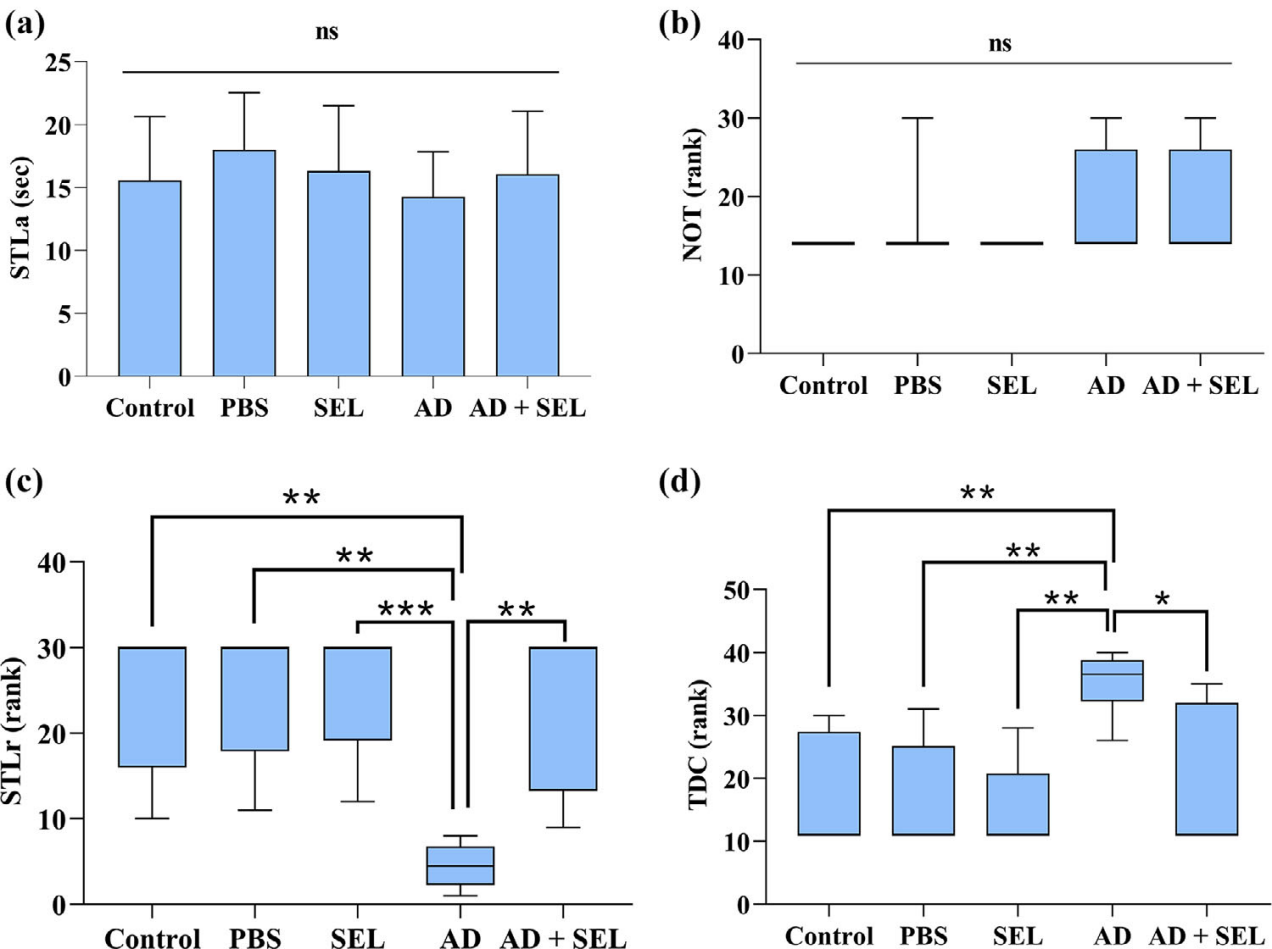
The effects of selegiline administration on passive avoidance learning (PAL) in the Aβ rats (*n* = 8). “a” represents step‐through latency in the acquisition trial (STLa) and presented as means ± SD (one‐way analysis of variance). “b,” “c,” and “d” represents the number of trials to reach learning (NOT), step‐through latency in the retention phase (STLr), and time spent in the dark compartment (TDC), respectively. Data are presented as the median interquartile range (Kruskal–Wallis test, Dunn's post hoc test). ns, no significance; *p < .05, **p < .01, and ***p < .001. AD, Alzheimer's disease; PBS, phosphate‐buffered saline; SEL, selegiline.

The one‐way analysis of variance did not show a significant difference in the results of the first‐day analysis for STLa (*F* (4, 35) = 0.66; *p* = .62; Figure 5a). Additionally, the comparison of the number of shocks received (NOT) on the first day did not show a significant difference (H _[4]_ = 4.45; *p* = .34; Figure 5b). The comparison of STLr results with the Kruskal–Wallis test showed a significant difference between groups (H _[4]_ = 22.16; *p* < .001; Figure 5c). Therefore, the AD group showed a significant decrease compared to the control group (*p* < .01). Additionally, the AD + SEL treatment group showed a significant increase compared to the AD group (*p* < .01). The comparison of TDC results with the Kruskal–Wallis test showed a significant difference between groups (H _[4]_ = 18.86; *p* < .001; Figure 5d). Therefore, the AD group showed a significant increase compared to the control group (*p* < .01). Additionally, the AD + SEL treatment group showed a significant decrease compared to the AD group (*p* < .05).

### 3.6 Effect of SEL and Aβ on TAC and TOS in AD rats

**FIGURE 6:**
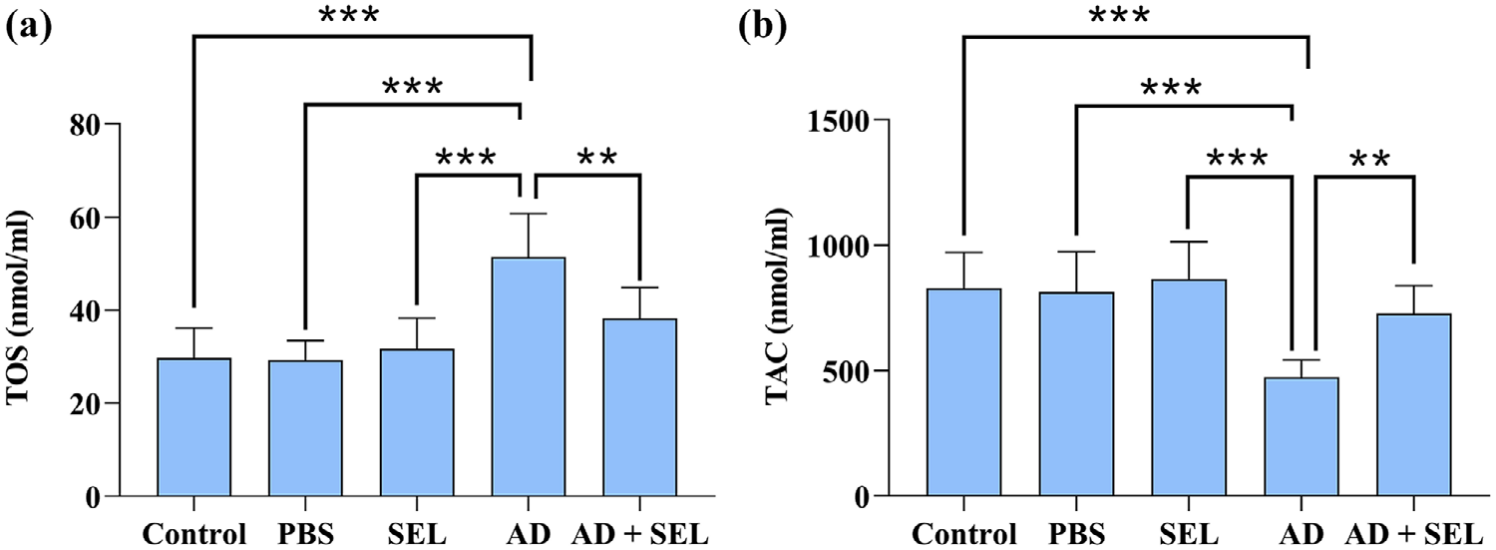
The effects of selegiline administration on the plasma parameters of total oxidant status (TOS) (a) and total antioxidant capacity (TAC) (b) of AD rats using assay kits. Data are presented as means ± SD of eight animals per group (one‐way analysis of variance, Tukey's post hoc test). **p < .01, ***p < .001. AD, Alzheimer's disease; PBS, phosphate‐buffered saline; SEL, selegiline.

The one‐way analysis of variance showed a significant difference in the plasma levels of TOS (*F* (4, 35) = 14.86; *p* < .001). Therefore, the AD group showed a significant increase compared to the control group (*p* < .001). Additionally, the AD + SEL treatment group showed a significant decrease compared to the AD group (*p* < .001; Figure 6a).

The one‐way analysis of variance showed a significant difference in the plasma levels of TAC (*F* (4, 35) = 11.80; *p* < .001). Therefore, the AD group showed a significant decrease compared to the control group (*p* < .001). Additionally, the AD + SEL treatment group showed a significant increase compared to the AD group (*p* < .01; Figure 6b).

## 4 DISCUSSION

The major findings of the present study are as follows: In AD rats, administration of SEL (1) modulated the oxidant/antioxidant status, (2) improved recognition memory in the NOR test, (3) ameliorated anxiety‐like behavior in the EPM test, (4) promoted spatial learning and memory in the MWM test, and (5) ameliorated the decline in passive avoidance memory.

In the present study, the locomotor activity of rats was evaluated using the OF test. The results showed that Aβ injection had no effect on the locomotor activity of rats, which is consistent with previous findings (Ahmadi et al., 2021). However, in line with previous studies (Zare et al., 2015), in the present study, Aβ injection significantly reduced the percentage of entries into open arms and time spent in open arms in the EPM test, indicating an increase in anxiety levels in the AD group. Interestingly, oral administration of SEL significantly reduced anxiety‐like behavior in the treated group. Similar findings have shown that SEL administration reduces anxiety‐like behavior in mice receiving methamphetamine (Gholami et al., 2023).

The effects of SEL treatment on cognitive memory were assessed using the NOR, PAL, and MWM tests in the present study. The results showed that Aβ injection led to impairment in cognitive memory in rats, which is consistent with previous studies (Ahmadi et al., 2021; Gholipour et al., 2022). According to the results of the present study, the time spent exploring the novel object and familiar object in the NOR test was almost the same in the AD group, indicating impairment in object recognition memory. Additionally, the time spent and distance traveled in the MWM task to find the hidden platform was significantly increased in the AD group compared to the control groups, indicating impairment in spatial memory. The time spent in the dark compartment of the shuttle box apparatus was significantly increased in the AD group compared to the control groups, indicating impairment in passive avoidance memory. In support of prior studies, in the present study, cognitive performance (object recognition memory, spatial memory, and passive avoidance memory) improved in rats treated with SEL. Previous studies showed that SEL treatment potentially improved cognitive activity by inhibiting lipid peroxidation, increasing intracellular antioxidant enzyme levels, and reducing acetylcholinesterase activity in the brain (Goverdhan et al., 2012). In another study, combined electroacupuncture treatment with selegiline improved cognitive performance due to inhibition of neuroinflammation and increased glucose metabolism in an animal model of AD (Cai & Yang, 2020). Consistent with the results of the present study, it has been shown that SEL can improve cognitive impairment in AD animal models (Pazini et al., 2013; Tsunekawa et al., 2008). Nevertheless, to the best of the authors’ knowledge, this is the first study reporting the protective properties of SEL against Aβ_1‐42_‐induced anxiogenic‐like behaviors.

Studies have clearly shown that oxidative stress is associated with anxiety (A. Komaki et al., 2016) and plays a key role in initiating neurological diseases such as AD (Briyal et al., 2023). Antioxidant factors have been shown to alleviate anxiety effectively (Salim et al., 2010) and subsequently improve cognitive dysfunction caused by AD (Ahmadi et al., 2021; Nazifi et al., 2021). In the present study, Aβ injection led to a decrease in TAC levels and an increase in TOS levels, disrupting the oxidative‐antioxidant balance in rat plasma. Existing evidence suggests that oxidative stress in neurodegenerative disorders, including AD, is not restricted to the central nervous system (Marcourakis et al., 2008). In this regard, increased peripheral oxidative stress in AD has been shown in previous experiments (Ahmadi et al., 2021; H. Komaki et al., 2019). Interestingly, treatment with SEL restored the balance by increasing TAC levels and suppressing the increase in TOS levels, indicating its antioxidant capacity. Previous studies have shown that selegiline is highly effective in reducing inflammatory parameters and oxidative stress caused by free radicals (Ahmed & Chetia, 2023). Furthermore, SEL has been shown to reduce levels of reactive oxygen species and malondialdehyde and increase superoxide dismutase activity in the liver of mice exposed to a high‐fat diet and physical activity (Tian et al., 2023). In a study, it has been reported that there is a negative correlation between oxidative stress factors and cognitive memory (S. Ghaderi et al., 2024). Studies have shown that neuroinflammation and the production of free radicals can lead to the destruction of neurons (Mancuso et al., 2007) and disruption in synaptic plasticity (Ahmadi et al., 2021), as the cellular basis of memory and learning, resulting in behavioral disorders. Additionally, improving the oxidative‐antioxidant status can prevent neuronal death and synaptic disturbances (Arabi et al., 2023). These results indicate that the ability of SEL to eliminate free radicals and prevent oxidative damage contributes to its protective effects against cognitive and behavioral impairments caused by Aβ and anxiety suppression.

## 5 CONCLUSIONS

In conclusion, oral treatment with SEL, by modulating the oxidative status, may improve anxiety and cognitive memory deficits in AD rats. This study suggests that SEL may be a promising agent against cognitive and non‐cognitive disturbances associated with AD. However, further research is needed to evaluate the mechanisms underlying the protective effects of SEL against AD‐induced cognitive impairment, especially the mechanisms involved in its antioxidant properties.

## AUTHOR CONTRIBUTIONS

**Behnam Mohamadpour**: Conceptualization; methodology; software; data curation; investigation; validation; formal analysis; visualization; writing—original draft; writing—review and editing. **Naser Mirazi**: Conceptualization; methodology; validation; supervision; funding acquisition; visualization; project administration; resources; writing—original draft; writing—review and editing. **Alireza Komaki**: Conceptualization; methodology; data curation; investigation; validation; supervision; funding acquisition; visualization; project administration; resources; writing—original draft; writing—review and editing. **Hamid Shokati Basir**: Methodology; software; data curation; investigation; validation; formal analysis; visualization; writing—original draft; writing—review and editing. **Abdolkarim Hosseini**: Methodology; software; data curation; validation; formal analysis; supervision; visualization; writing—original draft; writing—review and editing. All the authors read and approved the final manuscript.

## FUNDING INFORMATION

This study did not receive any external funding.

## CONFLICT OF INTEREST STATEMENT

The authors declare no conflicts of interest.

### PEER REVIEW

The peer review history for this article is available at https://publons.com/publon/10.1002/brb3.3599

## Data Availability

All data generated and analyzed during the current study are available with the corresponding author upon reasonable request.
